# USP9X-mediated KDM4C deubiquitination promotes lung cancer radioresistance by epigenetically inducing TGF-β2 transcription

**DOI:** 10.1038/s41418-021-00740-z

**Published:** 2021-02-08

**Authors:** Xiaohua Jie, William Pat Fong, Rui Zhou, Ye Zhao, Yingchao Zhao, Rui Meng, Sheng Zhang, Xiaorong Dong, Tao Zhang, Kunyu Yang, Gang Wu, Shuangbing Xu

**Affiliations:** Cancer Center, Union Hospital, Tongji Medical College, Huazhong University of Science and Technology, Wuhan, 430022 China

**Keywords:** Tumour biomarkers, Preclinical research

## Abstract

Radioresistance is regarded as the main barrier to effective radiotherapy in lung cancer. However, the underlying mechanisms of radioresistance remain elusive. Here, we show that lysine-specific demethylase 4C (KDM4C) is overexpressed and correlated with poor prognosis in lung cancer patients. We provide evidence that genetical or pharmacological inhibition of KDM4C impairs tumorigenesis and radioresistance in lung cancer in vitro and in vivo. Moreover, we uncover that KDM4C upregulates TGF-β2 expression by directly reducing H3K9me3 level at the TGF-β2 promoter and then activates Smad/ATM/Chk2 signaling to confer radioresistance in lung cancer. Using tandem affinity purification technology, we further identify deubiquitinase USP9X as a critical binding partner that deubiquitinates and stabilizes KDM4C. More importantly, depletion of USP9X impairs TGF-β2/Smad signaling and radioresistance by destabilizing KDM4C in lung cancer cells. Thus, our findings demonstrate that USP9X-mediated KDM4C deubiquitination activates TGF-β2/Smad signaling to promote radioresistance, suggesting that targeting KDM4C may be a promising radiosensitization strategy in the treatment of lung cancer.

## Introduction

Cancer is as much a genetic disease as it is an epigenetic disease, and epigenetic alterations including histone modifications and DNA methylation are involved in tumor development and progression [1]. Histone lysine demethylase 4C (KDM4C, also known as JMJD2C/GASC1) is a member of the JmjC-domain-containing protein family that encodes a histone demethylase for di- and trimethylated lysine 9 and 36 on histone 3 (H3K9me3/2 and H3K36me3/2) and has been shown to associate with malignant transformation [2, 3]. Accumulating evidence suggests that KDM4C is a candidate oncoprotein that is amplified or overexpressed in various cancer types [4–6]. In addition, several studies have demonstrated that KDM4C regulates the transcription of MYC, Il3ra and cyclin E1 by removing the repressive histone mark H3K9me3, which in turn initiate metabolic reprogramming, control B cell activation and promote cell growth [7–10]. Excitingly, recent studies have demonstrated that SD70 is a novel KDM4C inhibitor and revealed its potential in impeding tumor progression in prostate cancer and acute myeloid leukemia [11, 12]. However, the regulatory mechanisms and roles of KDM4C in cancer radioresistance have not yet been investigated.

Transforming growth factor-β (TGF-β) is a critical modulator of numerous cellular activities that are central to tumorigenesis, with the main regulating pathways being canonical Smad-dependent and noncanonical Smad-independent signaling [13]. The TGF-β/Smad signaling pathway has been pivotally implicated in many aspects of cellular processes, including tumor metastasis, immune regulation and microenvironment modification [14]. Importantly, the roles of TGF-β/Smad signaling in the DNA damage response have also been documented [15–17]. For instance, Smad2 and Smad7 are critical intermediaries between TGF-β and ataxia-telangiectasia mutated (ATM) crosstalk, while Smad3 binds to BRCA1 and inhibits BRCA1-dependent DNA damage repair [15, 16]. In addition, preclinical studies using LY2109761, a novel TGF-β receptor type I/II dual inhibitor, reported direct radiosensitizing effects in glioblastoma and breast cancer [18, 19]. All these studies strongly support that activation of TGF-β/Smad signaling is closely associated with cancer radioresistance.

In this study, we show that KDM4C is overproduced and predicts poor clinical outcomes in lung cancer patients. Furthermore, we present evidence that genetical or pharmacological inhibition of KDM4C overcomes radioresistance in lung cancer by inactivating the TGF-β2/Smad/ATM signaling pathway. Importantly, we are the first to report that the deubiquitinase USP9X is responsible for the upregulation of KDM4C in lung cancer. These data reveal that KDM4C is a promising therapeutic drug target and that its inhibitor SD70 is a potential radiosensitizer for lung cancer.

## Materials and methods

### Cell culture and plasmid transfection

All cell lines were purchased from American Type Culture Collection and cultured in either RPMI-1640 or DMEM supplemented with 10% FBS and 100 IU/mL Penicillin-Streptomycin solution at 37 °C in 5% CO_2_. All cell lines were tested for mycoplasma contamination and authenticated using short tandem repeat (STR) profiling. Polymerase chain reaction (PCR) was used to generate the KDM4C plasmid, which was subcloned into the pDNOR201 entry vector and subsequently transferred to a gateway-compatible destination vector for the addition of SFB tag to express SFB-KDM4C protein using Gateway Technology (Invitrogen). Lipofectamine 2000 reagent (Invitrogen) was used for all plasmid transfections.

### Antibodies and reagents

Several primary antibodies were used in this study: rabbit anti-KDM4A (Bethyl Laboratories, A300-861A, 1:1000), rabbit anti-KDM4B (Bethyl Laboratories, A301-478A, 1:1000), rabbit anti-KDM4C (Bethyl Laboratories, A300-885A, 1:800), rabbit anti-USP9X (Proteintech, 55054-1-AP, 1:1000), rabbit anti-HA (Cell Signaling Technology, #3724, 1:1000), rabbit anti-β-Catenin (Cell Signaling Technology, #8480, 1:1000), rabbit anti-phospho-Chk2 (Thr68, Cell Signaling Technology, #2197, 1:1000), rabbit anti-Smad2/3 (Cell Signaling Technology, #8685, 1:1000), rabbit anti-phospho-Smad2 (Ser465/Ser467, Cell Signaling Technology, #18338, 1:1000), rabbit anti-phospho-Smad3 (Ser423/425, Cell Signaling Technology, #9520, 1:1000), rabbit anti-TGF-β2 (Proteintech, 19999-1-AP, 1:1000), rabbit anti-TGFβR1 (ABclonal, A16983, 1:1000), rabbit anti-TGFβR2 (ABclonal, A1415, 1:1000), mouse anti-GAPDH (ABclonal, AC033, 1:3000), rabbit anti-ATM (ABclonal, A19650, 1:1000), rabbit anti-Chk2 (ABclonal, A19543, 1:1000), mouse anti-Flag (Sigma-Aldrich, F1804, 1:1000), rabbit anti-H3K9me3 (Abcam, Ab8898, 1:800), rabbit anti-H3K36me3 (Abcam, Ab9050, 1:800), rabbit anti-H3 (Abcam, Ab1791, 1:1000), rabbit anti-phospho-ATM (Ser1981, Abcam, Ab81292, 1:1000) and rabbit anti-Rad51 (Abcam, Ab133534, 1:1000). The reagents used were as follows: SD70 (Xcess Biosciences, M60194-b), recombinant human TGF-β2 (Abcam, ab84070), LY2109761 (Selleck, HY -12075), MG132 (Calbiochem, 474790) and cycloheximide (Sigma-Aldrich, A8244).

### RNA interference

SiRNA transfection was carried out using Lipofectamine RNAiMAX transfection reagent (Invitrogen) for 48 h. The following sequences of the siRNAs were used:

Scramble siRNA, 5′-UUCUCCGAACGUGUCACGU-3′;

SiKDM4C#1, 5′-AGAUAGCAGCAAUGAAGAA-3′ [20];

SiUSP9X, 5′-AGAAAUCGCUGGUAUAAAUUU-3′ [21–23];

SiTGF-β2 #1, 5′-GCAAUGGAGAAGAAUGCUU-3′ [24];

SiTGF-β2 #2, 5′-GGAUUGAGCUAUAUCAGAU-3′ [24].

### Establishment of stable lung cancer cell lines

Stable KDM4C-depleted cell lines were established as previously described [25, 26]. In brief, HEK293T cells were transfected with the indicated shRNAs, packaged plasmids psPAX2 and pMD2.G. After 48 h, the supernatants were harvested and filtered using a 0.45 μm filter. SPC-A1 and H460 cells were then infected using the filtered lentiviral supernatants with the addition of 10 μg/mL of polybrene (Sigma-Aldrich). Stable cells were screened using puromycin (2 μg/mL) and confirmed by Western blotting. The following sequences of the shRNAs were used:

Control shRNA: 5′-CTCGCTTGGGCGAGAGTAAG-3′;

KDM4C shRNA-1: 5′-GCAGAGAGTAATGGTGTGTTA-3′ [27];

KDM4C shRNA-2: 5′-TAGTGAATCGAACTTCTGG-3′.

### Tandem affinity purification S-Flag-SBP (SFB)-tagged KDM4C complexes

This technology was conducted as previously described [26, 28, 29]. In brief, HEK293T cells stably expressing exogenous SFB-KDM4C were harvested and lysed with NETN buffer (100 mM NaCl, 1 mM EDTA, 20 mM Tris-HCl (pH 8.0) and 0.5% Nonidet P-40) at 4 °C for 20 min. The lysates were centrifuged at 14000 *rpm* and incubated with streptavidin-conjugated beads (Amersham) at 4 °C for 2 h. Bead-bound proteins were washed and eluted twice using 2 mg/mL biotin (Sigma-Aldrich) diluted in NETN buffer for 2 h. The elutes were further incubated with S-protein agarose (Novagen) for 1 h. After being washed three times, the immunocomplexes were subjected to Western blotting. Protein bands were excised and further analyzed by mass spectrometry (performed by Taplin Mass Spectrometry Facility, Harvard Medical School).

### Western blotting and immunoprecipitation

Cell lysates were prepared using NETN buffer and resolved by SDS-PAGE for Western blotting. The supernatants were initially incubated overnight with S-protein agarose (Novagen) at 4 °C for exogenous immunoprecipitation. For endogenous binding, cell lysates were incubated with the anti-KDM4C or USP9X antibody at 4 °C for 1 h before protein A/G agarose (Santa Cruz Biotechnology) was added. Lysates were then washed with NETN buffer five times prior to Western blotting analysis.

### In vivo ubiquitination assay

Transfection of indicated siRNAs and corresponding plasmids into cells was carried out prior to the addition of 10 μM MG132 for 4 h prior to harvesting. Cells were lysed using NETN buffer solution. S-protein agarose (Novagen) was then added and put on a rotating incubator overnight at 4 °C. The beads were then washed five times, boiled with SDS loading buffer and subjected to Western blotting.

### RNA sequencing and analysis

Total RNA was extracted from cells using TRIzol reagent (Invitrogen). RNA sequencing (RNA-seq) was performed by CapitalBio Technology (Beijing, China). RNA integrity was verified using an Agilent 2100 Bioanalyzer (Agilent Technologies). Paired-end library sequencing with Illumina HiSeq 2500 was performed with the mRNA-seq sample prep kit (Illumina) according to the manufacturer’s specifications. The Illumina data analysis pipeline was then used for sequence analysis. All samples were allocated to lanes and processed in a blinded fashion to keep bias to a minimum. The RNA-seq data have been deposited in the Gene Expression Omnibus database under accession number GSE136404 (https://www.ncbi.nlm.nih.gov/geo).

### Real-time quantitative PCR

Extraction of total RNA from cell lysates was done using TRIzol reagent (Takara). cDNAs were synthesized using qPCR RT Master Mix (Toyobo, Osaka, Japan). Each PCR was performed in triplicate. The relative mRNA levels of target genes were determined by the ΔCt method (the Ct of GAPDH minus the Ct of the target gene). Primer sequences are given in Supplementary Table S1.

### Chromatin immunoprecipitation

ChIP assay was performed following the protocol of the EZ-ChIP^™^ Chromatin immune-precipitation kit (Millipore). The DNA pulled down by the anti-H3K9me3 or IgG antibody was amplified by PCR. The H3K9me3-bound DNA of TGF-β2, BMP2 and JUND gene promoters was quantified by real-time PCR in triplicates using gene promoter-specific primers. Data were normalized to the corresponding DNA input control. The specific primers are listed in Supplementary Table S2.

### CCK-8 assay

The half-maximal inhibitory concentrations (IC50s) of SD70 were analyzed using Cell count KIT-8 assay (CCK-8, Dojindo, Japan). Briefly, 1 × 10^3^ cells were seeded in 96-well plates and cultured for 24 h, 48 h and 72 h respectively, followed by incubation with 8 μl CCK-8 assay solution in each well for 1 h. The absorbance values at 450 nm were measured using a Multimode Plate Reader (EnSpire^®^ 2300, USA).

### Cell clonogenic survival assay

Equal number of SPC-A1 or H460 cells were plated in triplicate and grown in 6-well plates for each indicated irradiation (IR) dose. After exposure to IR, cells were cultured for ~2 weeks and washed with PBS buffer solution before fixation using methanol for 30 min. Crystal violet staining solution (0.5%) was used to dye cells. Colonies consisting of a nonoverlapping group of at least 50 cells were counted manually by a blinded reader using a light microscope. The single-hit multi-target model was used to calculate the surviving fraction, which was performed as previously described [30].

### Neutral comet assay

The neutral comet assay was performed in triplicate using the Comet Assay Kit (Trevigen, 4250-050-K) according to the manufacturer’s specifications. In brief, cells were irradiated at 6 Gy and collected after 4 h, after which they were immobilized on the comet slide using low melting agarose. Lysis was performed for 1 h and washed three times with neutral electrophoresis buffer before being subjected to electrophoresis at 4 °C for 1 h. Gels were then neutralized and stained using SYBR Gold (Invitrogen), and cells were viewed using a fluorescence microscope and analyzed using CometScore 2.0 and GraphPad Prism Software. Olive tail moments were analyzed using at least 100 cells from each group.

### Immunofluorescence staining

SPC-A1 or H460 cells transfected with KDM4C targeting shRNAs or treated with SD70 were seeded on coverslips and subjected to 6 Gy IR. At 4 h postirradiation, cells were fixed using 4% paraformaldehyde, permeabilized with 0.2% Triton X-100 for 15 min and then blocked with 5% bovine serum albumin. Subsequently, the samples were incubated with anti-phospho-ATM (Ser1981, 1:500), anti-phospho-Chk2 (Thr68, 1:500) or anti-Rad51 (1:500) antibodies overnight and secondary antibody for 1 h. Cells were counterstained with DAPI for 10 min and observation was done by fluorescence microscope.

### Animal experiments

All animal experiments were approved by The Medical Ethics Committee of Tongji Medical College, Huazhong University of Science and Technology. Four- to six-week-old BALB/c nude mice were allocated randomly into four groups (eight mice per group) and were injected subcutaneously with 5 × 10^6^ SPC-A1 stable cells (shControl, shKDM4C-1, shKDM4C-2). A radiation dose of 10 Gy was given when tumors reached a volume of 130 mm^3^.

For SD70 treatment, SD70 drug preparation and dosing for xenograft experiments were performed as described [11]. Briefly, nude BALB/c mice were allocated at random into four groups (eight mice per group), and lung tumor cells were implanted subcutaneously. SD70 was administered intraperitoneally at 10 mg/kg in PEG300/D5W starting from the first day when the tumor volume reached a calculated average of 130 mm^3^ and continued for 4 weeks with five consecutive injections in the first week and every other day for the next 3 weeks. Mice in the IR groups were subjected to 10 Gy local IR using an X-ray irradiator (Varian, USA) at a dose rate of 6 Gy/min on the day after the first injection of SD70 or vehicle, which was previously described [30]. All tumors were measured by a blinded reader using Vernier calipers every 3 days, and the formula used to calculate tumor volumes was as follows: V = (L × W^2^)/2, where V = volume (mm^3^), L = length (mm), and W = width (mm).

### Lung cancer tissue microarray and immunohistochemical (IHC) staining

Lung adenocarcinoma tissue microarrays containing lung carcinoma tissues and paired healthy lung tissues were provided by Outdo Biotech (Shanghai, China). IHC analysis was carried out as previously described [28, 29]. The percentage of the positive area and the staining intensity were both used to evaluate KDM4C and USP9X expression. The staining index (values 0–12) indicated the percentage of positively stained cells (0–25% = 1, 26–50% = 2, 51–75% = 3, >75% = 4) and the intensity of positive staining (negative = 0, weak = 1, moderate = 2, or strong = 3) and was calculated by multiplying these two values; a score <8 was defined as low expression, and a score equal to or higher than eight was deemed high expression.

### Statistics analysis

All in vitro experiments were carried out independently and in triplicate. All quantitative data are presented as the mean ± SD unless stated otherwise. All statistical power analysis for group comparisons were performed using Student’s *t* test (two-tailed). The Kaplan–Meier method was used to evaluate overall survival. Pathway enrichment analysis was performed using the PANTHER pathway database. *P* value < 0.05 were considered statistically significant (*P < 0.05, **P < 0.01, ***P < 0.001).

## Results

### KDM4C is overproduced and predicts poor clinical outcomes in lung cancer patients

**Fig. 1:**
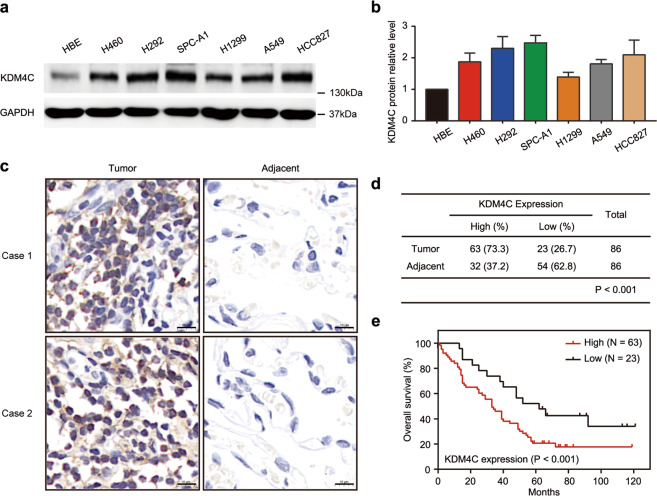
KDM4C is upregulated and predicts poor clinical outcomes in lung cancer. **a** KDM4C protein levels in different cell lines were examined by Western blotting. **b** Quantification of KDM4C protein levels in (**a**) (*n* = 3). **c** Representative IHC staining pictures for KDM4C in lung adenocarcinoma tissues and adjacent lung tissues. Scale bar, 10 μm. **d** Statistical analysis of IHC staining to assess KDM4C expression in a lung cancer tissue microarray. **e** High KDM4C expression is correlated with adverse overall survival in lung cancer patients.

To evaluate the clinical significance of KDM4C in lung cancer, we first detected the protein level of KDM4C in six human lung cancer cell lines and as predicted, KDM4C protein level was upregulated compared to that in normal human lung epithelial cell HBE (Fig. 1a, b). Next, using immunohistochemistry (IHC) analysis, we quantified KDM4C protein levels in a human lung cancer tissue microarray consisting of 86 fresh lung adenocarcinoma tissues matched with their adjacent nontumor tissues (NTs) and demonstrated that KDM4C localized to the nucleus and cytoplasm of cancer cells and its protein levels were considerably higher in lung cancer tissues than in adjacent NTs (Fig. 1c, d). Moreover, we showed that there was a correlation between high expression of KDM4C and shorter overall survival in lung cancer patients by Kaplan–Meier survival analysis (*P* < 0.05) (Fig. 1e). These data suggest that KDM4C is overexpressed and related to a worse prognosis in lung cancer.

### KDM4C silencing impairs tumorigenesis and enhances radiosensitivity in lung cancer in vitro and in vivo

**Fig. 2:**
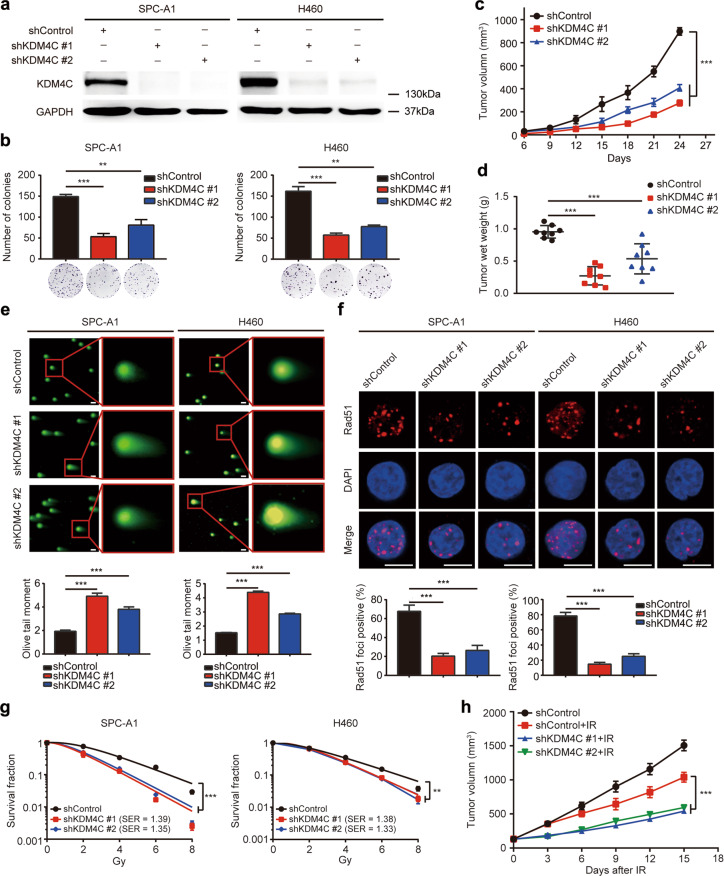
KDM4C depletion suppresses tumorigenesis and enhances radiosensitivity in lung cancer in vitro and in vivo. **a** SPC-A1 and H460 cells were transfected with the control or shKDM4C lentivirus, and samples were collected and analyzed by Western blotting using indicated antibodies (*n* = 3). **b** SPC-A1 and H460 cells stably expressing control or KDM4C shRNAs were seeded and cultured for 2 weeks. Crystal violet was then used to stain colonies which were counted under a microscope. **P < 0.01, ***P < 0.001 (*n* = 3). **c** 5 × 10^6^ SPC-A1 stable cells (Sh-Control or Sh-KDM4C) were subcutaneously injected into BALB/c nude mice. The growth curves of the xenograft tumors in the three groups are presented (*n* = 8 mice for each group). ***P < 0.001. **d** Tumor weights in three groups were shown (*n* = 8 mice for each group). ***P < 0.001. **e** Representative pictures of comet assays performed 4 h after IR exposure of KDM4C-depleted or control cells. ***P < 0.001 (*n* = 3). Scale bar, 20 μm. **f** KDM4C-depleted SPC-A1 and H460 cells were irradiated and harvested 4 h later. Upper panel: Representative immunostaining pictures of Rad51 foci. Lower panel: Rad51 foci quantification results. A cell containing 10 or more foci was considered as a foci-positive cell. ***P < 0.001 (*n* = 3). Scale bar, 10 μm. **g** KDM4C silencing led to enhanced radiosensitivity. Data are presented as the mean ± SD. SER: sensitization enhancement ratio. **P < 0.01, ***P < 0.001 (*n* = 3). **h** The growth curves of xenograft tumors for each group are represented. Data are presented as mean ± SEM (*n* = 8 mice for each group). *** *P* < 0.001.

Considering that KDM4C is substantially overexpressed in lung cancer tissues, we assumed that it might exert a tumorigenic effect in lung cancer. To this point, we first constructed KDM4C knockdown lung cancer cells using shRNAs (Fig. 2a) and found that loss of KDM4C inhibited cell proliferation (Fig. 2b). Subsequently, we inoculated T-cell-deficient athymic nude mice with KDM4C control and knockdown cells and showed that shKDM4C xenografts displayed significantly decreased tumor growth and tumor weight compared to control xenografts (Fig. 2c, d). These findings suggest that KDM4C silencing impairs tumorigenesis in lung cancer in vitro and in vivo.

It has been reported that the specific substrates of KDM4C, H3K9, and H3K36 are closely linked with double-strand break repair [31, 32], therefore we speculated that KDM4C might be involved in regulating radiosensitivity. To test this hypothesis, we first performed a neutral comet assay and found that the olive tail moment is higher in KDM4C deficient cells compared to that in control cells (Fig. 2e). In agreement with this idea, DNA damage-induced Rad51 foci was decreased in KDM4C knockdown cells (Fig. 2f). These results imply that inhibiting KDM4C accelerates DNA damage and impedes DNA repair. To further validate our findings, we used a clonogenic survival assay to determine the growth ability of cells after IR. As expected, loss of KDM4C enhanced cellular sensitivity to radiation (Fig. 2g). Next, we examined the effect of KDM4C knockdown in xenografts exposed to IR. As shown in Fig. 2h, the post-irradiation control group had a smaller tumor size compared to the non-exposed control group, indicating that IR was effective. More importantly, the post-IR shKDM4C group showed an even more significant decrease in tumor size compared to the post-IR control group (Fig. 2h). These results suggest that targeting KDM4C impedes tumorigenesis and renders lung cancer cells more susceptible to IR in vitro and in vivo.

### Inhibition of KDM4C with a pharmacologic drug SD70 hinders lung cancer progression and enhances response to radiotherapy in vitro and in vivo

**Fig. 3:**
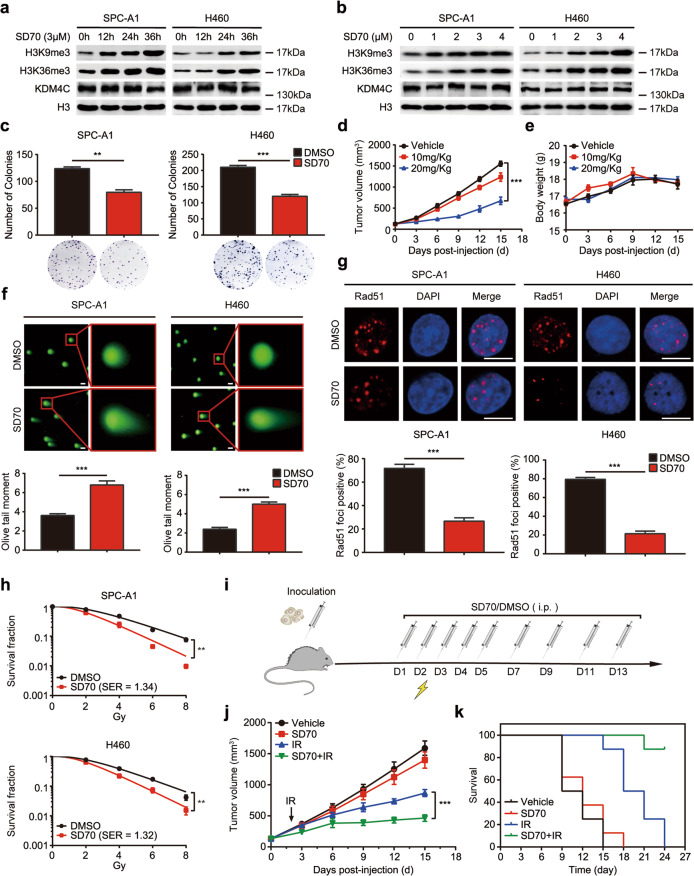
Inhibition of KDM4C with SD70 impairs cell proliferation and radioresistance in lung cancer in vitro and in vivo. **a**, **b** The inhibition efficacy of SD70 is time and concentration-dependent (*n* = 3). **c** SPC-A1 and H460 cells were treated with SD70 (3 μM) for 24 h and 48 h, respectively. Cells were then seeded and cultured for 2 weeks. The colonies were stained using crystal violet solution and then counted. Representative pictures are shown. **P < 0.01, ***P < 0.001 (*n* = 3). **d** Inhibition of KDM4C using indicated doses of SD70 impairs tumor growth in lung cancer in vivo (*n* = 5 mice for each group). ***P < 0.001. **e** Changes in body weight of xenografts post-inoculation were represented. **f** Neutral comet assay shows the effect of SD70 treatment on tail formation. The olive tail moment was quantified and graphed. ***P < 0.001 (*n* = 3). Scale bar, 20 μm. **g** Quantification of Rad51 foci intensity in SPC-A1 and H460 cells after treatment with SD70. Representative images are shown. Scale bar, 10 μm. ***P < 0.001 (*n* = 3). **h** The effects of SD70 treatment on the radiosensitivity of lung cancer cells were determined by clonogenic survival assay. **P < 0.01, ***P < 0.001 (*n* = 3). **i** Schematic representation of the xenograft study design and experimental workflow. **j** Growth curves of xenograft tumors in each group. Mice were treated with SD70 at the concentration of 10 mg/kg (*n* = 8 mice for each group). ***P < 0.001. **k** Kaplan–Meier analysis of mice survival due to treatments in (**j**).

To further evaluate the therapeutic potential of KDM4C inhibition, we used SD70, a selective competitive inhibitor of KMD4C, to block its catalytic domain without affecting its expression and tested its efficacy on lung cancer cell functions. Using CCK-8 assay, we determined the half- maximal inhibitory concentrations (IC50s) of SD70 in SPC-A1 (24 h) and H460 (48 h) cell lines, and we used 3 μM SD70 for all subsequent experiments (Supplementary Fig. 1). As shown in Fig. 3a and b, the accumulation of both H3K9me3 and H3K36me3 was time and concentration-dependent when cells were treated with SD70, while KDM4C levels remained constant, validating that SD70 can indeed inhibit the activity of KDM4C. Furthermore, we found that SD70 was able to suppress lung cancer cell proliferation in vitro (Fig. 3c). Consistent with our in vitro results, KDM4C inhibition effectively hindered tumor growth in xenografts in a concentration-dependent manner (Fig. 3d). Importantly, the weight of nude mice remained constant, implying that SD70 is non-toxic and safe (Fig. 3e). In addition, we also evaluated the potential pharmacological effects of SD70 in radiosensitization. As shown in Fig. 3f and g, increased olive tail moment and impaired Rad51 foci formation were observed when cells were treated with SD70. In line with these notions, SD70 treatment enhanced cell radiosensitivity to IR (Fig. 3h). Next, we assessed the pharmacological efficacy of SD70 in vivo using the experimental workflow (Fig. 3i). The growth of tumor xenografts was slightly impaired when inoculated nude mice were treated with 10 mg/kg SD70 (Fig. 3j). However, when xenografts were exposed to a combination of SD70 (10 mg/kg) and IR, the tumor growth was considerably subdued compared to the group exposed to IR only (Fig. 3j). Moreover, mice from the combined treatment group had a strikingly better overall survival (Fig. 3k). These results provide preclinical evidence for SD70 as a conceivable therapeutic drug in the treatment of lung cancer, especially as a radiosensitizer.

### KDM4C induces the expression of TGF-β2 and then activates Smad signaling by removing the repressive mark H3K9me3 at the TGF-β2 promoter

**Fig. 4:**
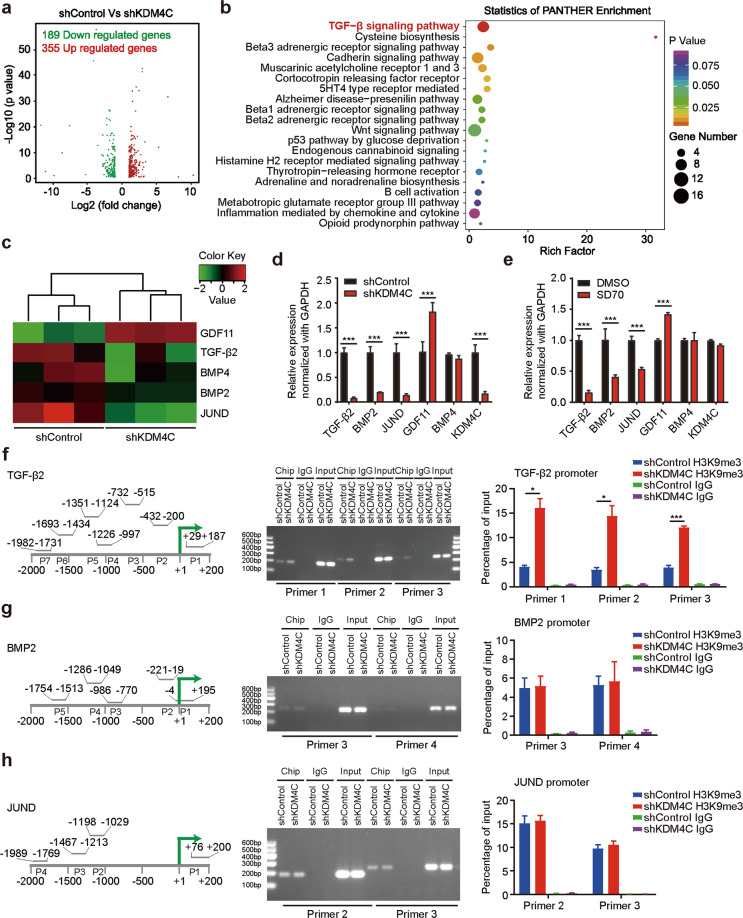
KDM4C prevents H3K9me3 accumulation at the TGF-β2 promoter to activate the TGF-β/Smad signaling pathway. **a** Volcano plot comparing control and KDM4C knockdown SPC-A1 cells. **b** Enrichment of differentially expressed genes in signaling pathways is illustrated in an advanced bubble chart. *Y*-axis represents pathways, and the *X*-axis represents rich factor. Size and color of the bubble are representation of the amount of differentially expressed genes enriched in pathways and their enrichment significance, respectively. **c** Heat map produced from RNA sequencing. **d**, **e** mRNA levels of indicated genes were measured using quantitative real-time PCR. ***P < 0.001 (*n* = 3). **f**–**h** An H3K9me3 ChIP assay was performed in shKDM4C and shControl cells. Left panel: ChIP primers were designed spanning from −2000 to +200 bp around the transcription start sites of indicated genes. Middle panel: Representative images of gel electrophoresis. Right panel: The H3K9me3 level in the gene promoter region was quantified and normalized to the input. IgG was used as the negative control. *P < 0.05; **P < 0.01; ***P < 0.001 (*n* = 3).

To identify the molecular pathways by which KDM4C exerts its biological effects in lung cancer, we employed RNA sequencing (RNA-Seq) screening and identified the expression of ~544 genes with significant changes (*P* value < 0.05 and foldchange absolute value ≥ 2) in KDM4C-depleted cells compared to that in control cells (Fig. 4a). Further pathway analysis demonstrated that the TGF-β signaling was significantly altered (Fig. 4b), indicating KDM4C may activate the TGF-β signaling pathway. RNA-seq results showed that a total of seven differentially expressed genes with more than twofold change were involved in the TGF-β signaling pathway (Fig. 4c). Several candidate genes were further validated. As illustrated in Fig. 4d and e, inhibition of KDM4C with shRNAs or SD70 treatment reduced the basal expression levels of TGF-β2, BMP2 and JUND while increasing the expression level of GDF11 in lung cancer cells. It has been well established that KDM4C-mediated reduction of H3K9me3 level often leads to transcriptional activation of target genes [2]. To determine whether KDM4C could directly regulate the H3K9me3 levels at the promoter of TGF-β2, BMP2 and JUND, we performed ChIP-qPCR analysis and revealed that KDM4C silencing caused substantial increases in the level of H3K9me3 at the TGF-β2 promoter, but not BMP2 and JUND promoters (Fig. 4f-h and Supplementary Fig. 2). These results indicate that KDM4C is needed for the transcription of TGF-β2 in lung cancer cells.

**Fig. 5:**
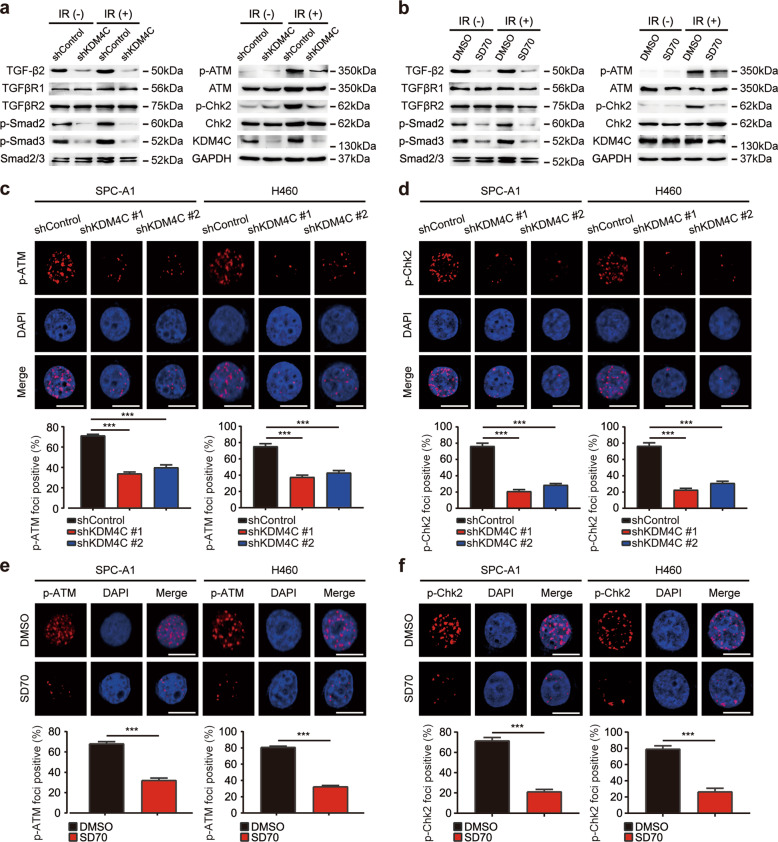
KDM4C is a key activator of the TGF-β2/Smad/ATM signaling pathway. **a**, **b** Changes in essential components of the TGF-β signaling pathway after genetical or pharmacological inhibition of KDM4C in SPC-A1 cells (*n* = 3). **c**, **d** SPC-A1 cells stably expressing control or KDM4C shRNAs were exposed to irradiation and collected 4 h later. Immunostaining was performed to examine p-ATM and p-Chk2 foci formation. ***P < 0.001 (*n* = 3). Scale bar, 10 μm. **e**, **f** SPC-A1 cells were treated with SD70 (3 μM) for 24 h and 48 h, respectively prior to exposure to irradiation. Cells were harvested after 4 h and subjected to immunostaining using p-ATM and p-Chk2 antibodies. Foci formation was quantified under a fluorescent microscope. ***P < 0.001 (*n* = 3). Scale bar, 10 μm.

Given that KDM4C positively controls the expression of TGF-β2, we speculated that KDM4C may be a key activator of TGF-β2/Smad signaling. To test this idea, we depleted KDM4C using shRNAs and found that TGF-β2, phosphorylated Smad2 and Smad 3 levels (p-Smad2 and p-Smad3) were decreased while TGFβR1, TGFβR2 and total Smad 2/3 remained constant when KDM4C was knocked down (Fig. 5a). Consistently, we observed similar results when KDM4C was inhibited using SD70 (Fig. 5b). In addition, it has been reported that TGF-β is necessary for DNA double-strand breaks recognition and regulates the kinase activity of ATM [16, 17], we therefore surmised that KDM4C might be involved in the activation of ATM signaling. To that end, we detected molecular changes of the ATM pathway and uncovered that both phosphorylated ATM and Chk2 levels were decreased while total ATM and Chk2 remained constant in response to DNA damage when KDM4C was inhibited (Fig. 5a, b). In line with the above results, DNA damage induced phospho-ATM and phospho-Chk2 foci formation were also impaired when KDM4C was blocked by shRNAs or SD70 (Fig. 5c-f). These data indicate that KDM4C stimulates TGF-β2/Smad signaling to activate the downstream ATM/Chk2 pathway in lung cancer.

### Tumor growth suppression and enhanced radiosensitivity caused by KDM4C silencing are dependent on the TGF-β2/Smad signaling pathway inactivation

**Fig. 6:**
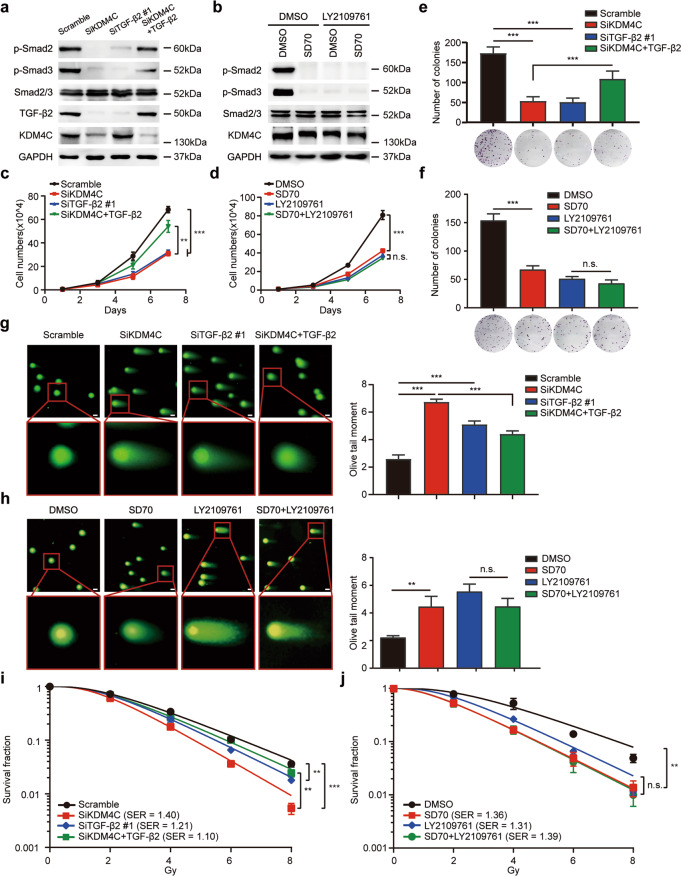
The TGF-β2/Smad signaling pathway activation is required for KDM4C-mediated effects on tumor growth and radioresistance in lung cancer. **a** SPC-A1 cells transfected with indicated siRNAs in the absence or presence of 4 ng/ml TGF-β2 for 24 h were collected and analyzed by Western blotting using indicated antibodies (*n* = 3). **b** SPC-A1 cells treated with LY2109761 (10 μM), SD70 (3 μM) or a combination of both were collected and analyzed by immunoblotting (*n* = 3). **c, d** SPC-A1 cells treated with indicated siRNAs or drugs were seeded at low density in 6-well plates. Cell numbers were counted every other day. **P < 0.01, ***P < 0.001 (*n* = 3). **e**, **f** SPC-A1 cells treat**e**d with indicated siRNAs or drugs were plated in triplicates. Histograms representing the numbers of colonies are depicted. ***P < 0.001 (*n* = 3). n. s. indicates no statistically significant difference (*P* > 0.05, *n* = 3). **g**, **h** Neutral comet assay performed after exposure to irradiation. Left panel: Representative images show comet tail. Scale bar, 10 μm. Right panel: the olive tail moment was quantified and graphed for each group. ** *P* < 0.01, ***P < 0.001 (*n* = 3). **i**, **j** SPC-A1 cells treated with indicated siRNAs or drugs were irradiated using indicated doses. Colonies containing more than 50 cells were recorded after 2 weeks. **P < 0.01, ***P < 0.001 (*n* = 3).

To confirm the involvement of the TGF-β2/Smad signaling pathway in KDM4C mediated effects in lung cancer, KDM4C and TGF-β2 were silenced with siRNAs and recombinant human TGF-β2 was added into KDM4C-depleted lung cancer cells. As shown in Fig. 6a, c, e and Supplementary Fig. 3, knockdown of TGF-β2 reduced the expression levels of p-Smad2 and p-Smad3 as well as suppressed lung cancer cell growth and proliferation whereas exogenous TGF-β2 elicited the opposite effects, suggesting that TGF-β2 activates Smad signaling and plays an oncogenic role in lung cancer. More importantly, the addition of recombinant human TGF-β2 partially rescued the declined levels of p-Smad2 and p-Smad3 in KDM4C-depleted cells (Fig. 6a). Functional experiments demonstrated that exogenous TGF-β2 partially reversed the inhibition of cell growth and the increase of radiosensitivity caused by KDM4C knockdown (Fig. 6c, e, g and i). These data reveal that KDM4C promotes lung cancer cell proliferation and radioresistance in a TGF-β2/Smad-dependent manner. To further investigate whether the TGF-β2/Smad pathway is required for the biological functions of KDM4C in lung cancer, cells blocked pharmacologically using SD70 were simultaneously treated with LY2109761, a novel TGF-β receptor type I/II dual inhibitor which inhibits the activation of the TGF-β2/Smad signaling pathway (Fig. 6b). As shown in Fig. 6d, f, h and j, the addition of LY2109761 inhibited proliferation and resulted in increased olive tail moment and sensitivity to IR, which is consistent with its reported actions. Strikingly, we found that further inhibition of KDM4C using pharmacological blockade has minimal effect on cell growth and radiosensitivity when cells were simultaneously treated with LY2109761 (Fig. 6 d, f, h and j). Together, our results indicate that KDM4C exerts its biological functions mainly via its ability to activate the TGF-β2/Smad signaling pathway.

### The deubiquitinase USP9X binds to the JmjN domain of KDM4C to prevent its degradation

**Fig. 7:**
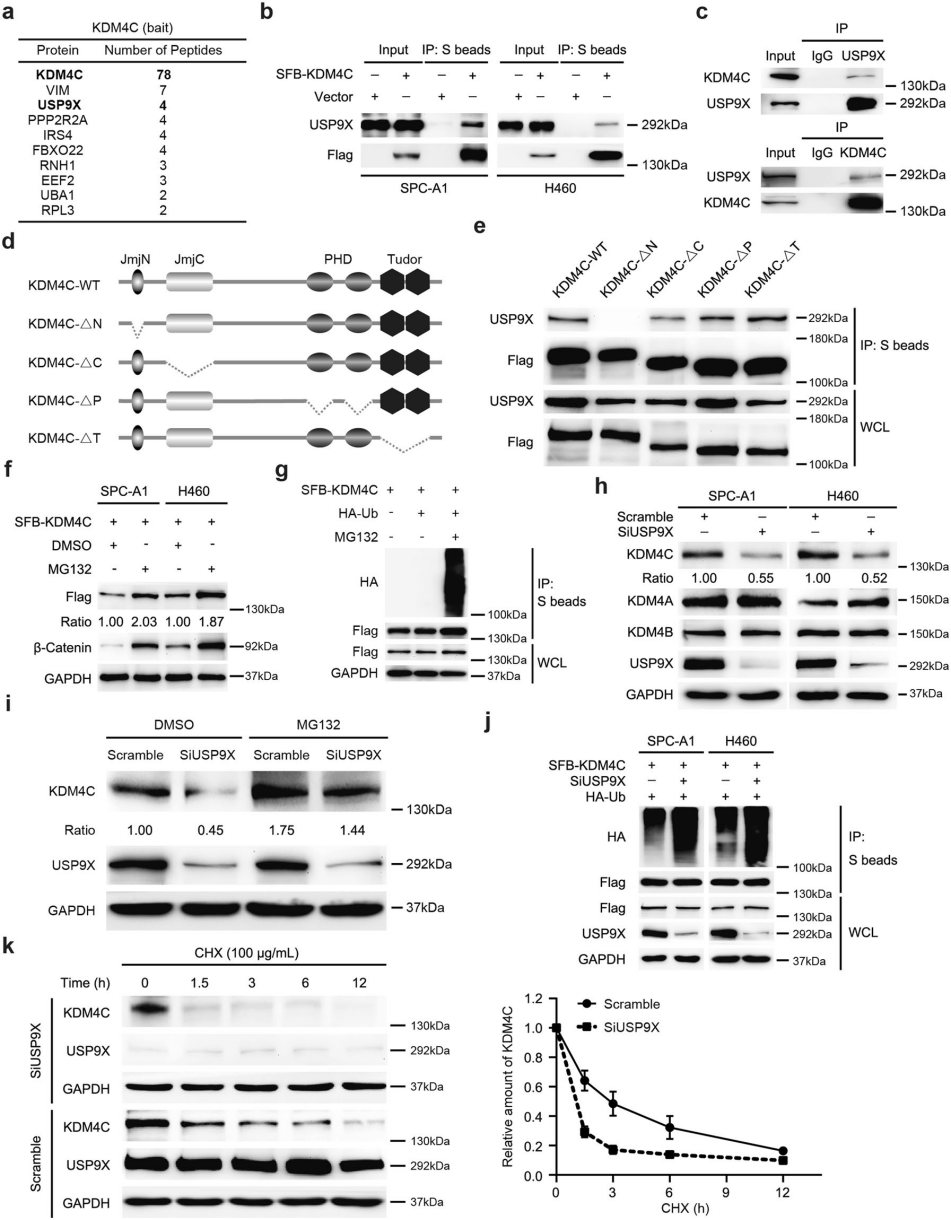
The deubiquitinase USP9X binds to the JmjN domain of KDM4C to prevent its degradation. **a** TAP-MS analysis shows KDM4C interacting proteins in HEK293T cells. The name and number of peptides for each protein identified are listed. **b** SPC-A1 and H460 cells were transfected with SFB-KDM4C plasmids. 24 h later, cells were harvested and then incubated with S-protein agarose prior to analysis by Western blotting (*n* = 3). **c** Endogenous interaction between KDM4C and USP9X in SPC-A1 cells (*n* = 3). **d** Schematic description of the domains of KDM4C and the generated deletion mutants. **e** The JmjN domain of KDM4C is required for its binding to USP9X. S protein beads were used for immunoprecipitation assay and cell lysates were subjected to immunoblotting (*n* = 3). WCL: whole cell lysate. **f** SPC-A1 and H460 cells were transfected with a plasmid encoding KDM4C for 24 h and then incubated with MG132 (10 μM) for 4 h prior to harvesting (*n* = 3). **g** SPC-A1 cells were transfected with the indicated constructs for 24 h, and MG132 (10 μM) was added for another 4 h. Cell lysates were then subjected to immunoprecipitation using S-protein beads and subsequent analysis by Western blotting (*n* = 3). **h** USP9X knockdown results in reduced levels of endogenous KDM4C (*n* = 3). **i** SPC-A1 cells were transfected with the indicated siRNAs for 48 h, incubated with DMSO or 10 μM MG132 for another 4 h. Cells were collected and probed for the indicated proteins by Western blotting (*n* = 3). **j** Cells were transfected with the indicated siRNAs and plasmids prior to treatment with MG132 (10 μM) for 4 h before collection. The lysates were incubated with S beads overnight and then subjected to immunoblotting (*n* = 3). **k** Left panel: SPC-A1 cells transfected with the indicated siRNAs for 48 h were treated with 100 μg/mL of cycloheximide (CHX) and collected at the indicated time points. Immunoblotting was performed to examine the protein level of KDM4C. Right panel: quantification of the KDM4C band intensity (*n* = 3).

To explore how KDM4C is upregulated in lung cancer, unbiased tandem affinity purification (TAP) and mass spectrometry (MS) proteomic analysis were conducted to identify KDM4C-interacting proteins. Excitingly, the deubiquitinase USP9X was one of the potential KDM4C binding proteins (Fig. 7a). To confirm this result, we carried out coimmunoprecipitation assays and found that exogenously transfected SFB-tagged KDM4C was able to form a complex with USP9X (Fig. 7b). In addition, we also showed the presence of an endogenous interaction between USP9X and KDM4C in lung cancer cells (Fig. 7c). Next, to map the domains of KDM4C responsible for binding to USP9X, we constructed a series of KDM4C deletion mutants and found that the JmjN domain deletion mutant failed to form a complex with USP9X, indicating that the JmjN domain of KDM4C binds to USP9X (Fig. 7d, e). Collectively, these results confirm that USP9X physically and specifically interacts with KDM4C in lung cancer cells.

USP9X is a deubiquitinase that has the ability to stabilize the ubiquitination substrate [21–23], therefore, we speculated that USP9X might affect the stability of KDM4C via the ubiquitin/proteasome pathway. Indeed, we observed marked protein accumulation and increased polyubiquitination of KDM4C after treatment with the proteasome inhibitor MG132 (Fig. 7f, g), indicating that KDM4C can be degraded by the proteasome. Moreover, depletion of USP9X with specific siRNAs, which have been described by other groups [21–23], resulted in a downregulation of KDM4C, while KDM4A and KDM4B remained unchanged (Fig. 7h). More importantly, we found that MG132 treatment substantially rescued the decline in KDM4C protein caused by USP9X depletion in lung cancer cells (Fig. 7i), implying that USP9X protects KDM4C from degradation by the proteasome. Furthermore, we demonstrated that USP9X silencing increased the polyubiquitination and shortened the half-life of KDM4C (Fig. 7j, k). These data provide substantial evidence that USP9X stabilizes KDM4C by preventing its degradation.

### USP9X silencing impairs TGF-β/Smad signaling and radioresistance by destabilizing KDM4C in lung cancer cells

**Fig. 8:**
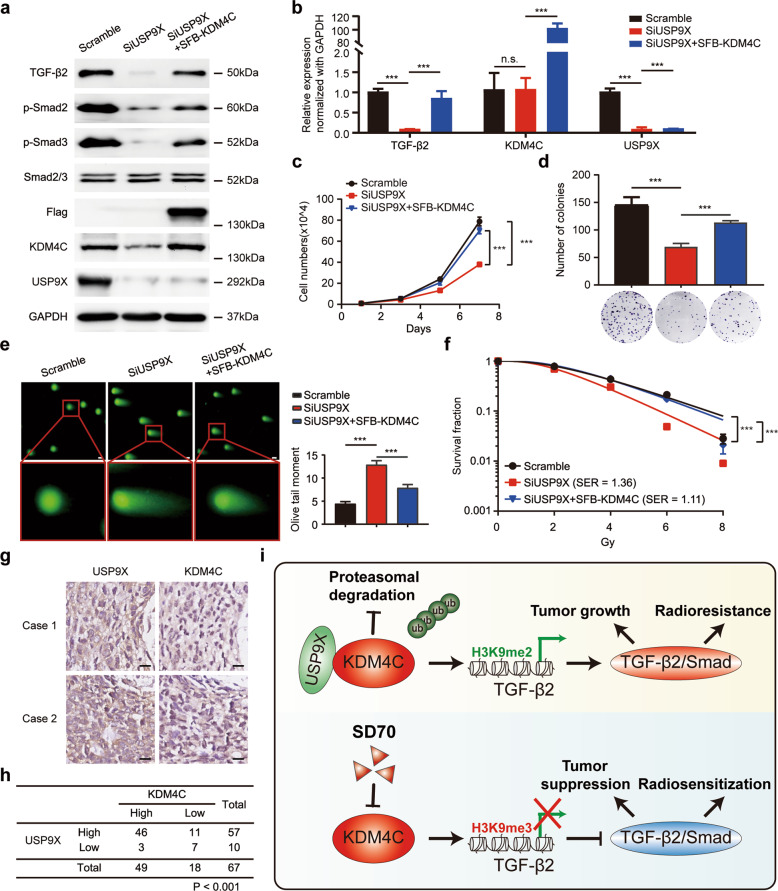
USP9X promotes tumor progression and radioresistance by stabilizing KDM4C in lung cancer. **a** SPC-A1 cells transfected with indicated siRNAs or SFB-KDM4C plasmid were collected and analyzed by Western blotting using indicated antibodies (*n* = 3). **b** SPC-A1 cells were transfected with indicated siRNAs or SFB-KDM4C plasmid, followed by real-time PCR to determine the mRNA levels of TGF-β2, KDM4C and USP9X. ***P < 0.001 (*n* = 3). **c** SPC-A1 cells transfected with the indicated siRNAs and plasmids were seeded and calculated every other day. Data are shown as the mean ± SD. ***P < 0.001 (*n* = 3). **d** SPC-A1 cells transfected with the indicated siRNAs and plasmids were seeded and cultured for 2 weeks. The colonies were stained using crystal violet solution and then counted under a microscope. ***P < 0.001 (*n* = 3). **e** Representative pictures of Comet assays performed 4 h after IR. ***P < 0.001 (*n* = 3). Scale bar, 20 μm. **f** SPC-A1 cells transfected with the indicated siRNAs and plasmids were irradiated with indicated doses. The percentages of surviving colonies were evaluated 2 weeks later. ***P < 0.001 (*n* = 3). **g** Representative immunohistochemical staining for USP9X and KDM4C in lung cancer tissues. Scale bar, 10 μm. **h** Positive correlation between USP9X and KDM4C protein levels in lung cancer tissues (*P* < 0.001, chi-square test). **i** Schematic diagram summarizes aberrant epigenetic networks and therapeutic potentials of targeting KDM4C in lung cancer. Upper panel: Deubiquitinase USP9X-mediated aberrant recruitment of KDM4C activates TGF-β2/Smad signaling to promote lung cancer progression and radioresistance. Lower panel: Pharmacological targeting of KDM4C using SD70 inhibits the activation of TGF-β2/Smad signaling, which in turn leads to enhanced radiosensitization.

To further clarify the role of USP9X in lung cancer radioresistance and confirm whether this effect is dependent on KDM4C, we transfected exogenously expressed KDM4C into USP9X-depleted cells and found that TGF-β2, p-Smad2 and p-Smad3 protein levels were decreased when USP9X was knocked down (Fig. 8a). In addition, downregulation of USP9X also led to reduced levels of TGF-β2 mRNA in lung cancer cells (Fig. 8b). Importantly, restoration of KDM4C partially rescued the reduced levels of TGF-β2, p-Smad2 and p-Smad3 in USP9X-depleted lung cancer cells (Fig. 8a, b), suggesting that USP9X silencing inhibits TGF-β2/Smad signaling in a KDM4C-dependent manner. We further observed that loss of USP9X suppressed the growth and proliferation of lung cancer cells, and these defects were partially restored by overexpression of KDM4C (Fig. 8c, d). In addition, our data also showed that USP9X-depleted lung cancer cells exhibited increased olive tail moment and enhanced radiosensitivity, and these phenotypes were also partially rescued by re-expressing KDM4C (Fig. 8e, f). Together, these results indicate that USP9X activates TGF-β2/Smad signaling and promotes radioresistance by stabilizing KDM4C in lung cancer cells.

## Discussion

In this study, we provide substantial evidence that supports the novel role of KDM4C in promoting radioresistance in lung cancer. Targeting of KDM4C with genetical or pharmacological inhibition significantly overcomes lung cancer radioresistance in vitro and in vivo. Furthermore, we provide new insights into the potency and efficacy of the epigenetic drug SD70 as a radiosensitizer in the treatment of lung cancer. Importantly, we identify the deubiquitinase USP9X as a novel upstream regulator which interacts with and stabilizes KDM4C and reveal that KDM4C-mediated H3K9me3 removal at the TGF-β2 promoter activates TGF-β2/Smad/ATM signaling. To our knowledge, this is the first report uncovering the implication of KDM4C in the regulation of lung cancer radioresistance.

The ubiquitin-proteasome system is the major regulator of intracellular protein degradation and turnover, and dysregulation of this system can result in overexpression of oncoproteins or downregulation of tumor suppressors, eventually leading to tumorigenesis [33]. Notably, the ubiquitination modification is a dynamic and reversible process since deubiquitinating enzymes (DUBs) are responsible for the specific removal of ubiquitin from proteolysis-targeted proteins and contribute to its stability [33]. Available evidence shows that KDM4C may act as an oncoprotein, however, the underlying upstream regulatory mechanisms remain elusive. We are first to uncover that deubiquitinase USP9X is a novel specific upstream modulator that controls the stability of KDM4C. USP9X, also called FAM, is frequently dysregulated in multiple cancers and plays an oncogenic role in lung cancer progression [22, 34–36]. In our study, we found that USP9X is highly expressed and correlated with poor overall survival in lung cancer patients (Supplementary Fig. 4). Moreover, we also uncovered that USP9X preferentially upregulates the protein levels of KDM4C by preventing its ubiquitination in lung cancer. This is the first report to reveal that KDM4C can be regulated and deubiquitinated at the posttranslational level. In addition, we revealed that USP9X is involved in lung cancer progression and radioresistance in a KDM4C-dependent manner. Given that USP9X co-localized with KDM4C and a significant positive correlation between the expression of USP9X and KDM4C in lung cancer tissues was observed (Fig. 8g, h and Supplementary Fig. 5), we conclude that USP9X overexpression may account for KDM4C upregulation in human lung cancer. Thus, our results strongly support a model that KDM4C is a novel substrate of deubiquitinase USP9X and its overproduction is the result of the deubiquitination modification by USP9X.

Aberrant expression of KDM4C has been observed in several types of cancers [5, 6], indicating its oncogenic roles in tumorigenesis. Our clinical data show that KDM4C is overexpressed and associated with poor prognosis in lung cancer. In addition, the functional results reveal that genetic deletion or pharmacological inhibition of KDM4C suppressed tumor growth in vitro and in vivo, further supporting our hypothesis that KDM4C acts as an oncoprotein in lung cancer. More importantly, we uncover a novel role of KDM4C in promoting lung cancer radioresistance. Our results show that KDM4C inhibition brought about increased DNA damage and subdued DNA repair response, rendering lung cancer cells and xenograft tumors more susceptible to IR. Mechanistically, we reveal that KDM4C activates TGF-β2/Smad signaling by promoting the removal of H3K9me3 at the TGF-β2 promoter. KDM4C inhibition reduces the expression of TGF-β2 and decreased the protein levels of p-Smad2 and p-Smad3 in lung cancer cells. In addition, our findings show that KDM4C activates TGF-β2/Smad signaling and its downstream ATM/Chk2 pathway, which may explain the mechanism behind increased radiosensitivity after KDM4C inhibition since Smad2 is a critical intermediary between the TGF-β and ATM signaling crosstalk essential in DNA repair [16, 17]. Therefore, these data support a notion that USP9X-mediated deubiquitination of KDM4C stimulates the activation of the TGF-β2/Smad/ATM signaling, and eventually contributes to radioresistance in lung cancer.

Epigenetic drugs such as histone deacetylase inhibitors and DNA-methyltransferase inhibitors have been widely used in lymphohematopoietic cancer therapy [37]. SD70, a selective inhibitor of KDM4C, is a newly developed epigenetic drug. We for the first time uncovered the roles of SD70 as an anti-cancer agent and radiosensitizer in lung cancer. Inhibition of KDM4C with SD70 can not only suppress cell growth in vitro and in vivo but also accelerate DNA damage and inhibit DNA repair, ultimately enhancing radiosensitivity in lung cancer. In our xenograft mouse model, combined treatment with relatively low SD70 concentration and IR exhibited a strong synergistic inhibitory effect on lung cancer. Thus, our findings provide a rationale for future clinical trials of combined treatment with SD70 and radiotherapy in lung cancer patients.

In summary, our study identifies the upstream modulator and downstream mediator of KDM4C and reveals that targeting KDM4C via blocking TGF-β2/Smad signaling impairs tumorigenesis and enhances radiosensitivity in lung cancer (Fig. 8i). More importantly, we provide preclinical evidence of the potency of SD70 as an attractive anti-cancer agent and radiosensitizer that may bring about prolonged overall survival in lung cancer patients.

## Supplementary information

Supplemental Material

Supplementary Figure 1

Supplementary Figure 2

Supplementary Figure 3

Supplementary Figure 4

Supplementary Figure 5
