# Supplementary material for: USP9X-mediated KDM4C deubiquitination promotes lung cancer radioresistance by epigenetically inducing TGF-β2 transcription
Source: Cell Death Differ. 2021 Feb 8;28(7):2095–111. doi: 10.1038/s41418-021-00740-z (PMC8257660; doi:10.1038/s41418-021-00740-z)
Supplement: Supplementary file 1 — Supplemental Material [file 41418_2021_740_MOESM1_ESM.docx]

**Supplementary Fig. 1 Determination of IC50 values by CCK8.** The IC50 values were obtained from at least three independent experiments.

**Supplementary Fig. 2 Screening and verification of chip primers.** Indicated gene promoter sequences in both input and recovered DNA immunocomplexes were detected by PCR and analyzed by gel electrophoresis.

**Supplementary Fig. 3 TGF-β2 activates Smad signaling to promote lung cancer cell growth and proliferation. a** SPC-A1 cells transfected with indicated TGF-β2 siRNAs in the absence or presence of IR were collected and analyzed by Western blotting using indicated antibodies (n = 3). **b** Western blotting of indicated proteins obtained from SPC-A1 cells treated with or without 4 ng/ml TGF-β2 for 24 h in the absence or presence of IR (n = 3). **c** SPC-A1 cells treated with or without 4 ng/ml TGF-β2 for 24 h were seeded and calculated every other day. Data are shown as the mean ± SD. *** P < 0.001 (n = 3). **d** SPC-A1 cells treated with or without 4 ng/ml TGF-β2 for 24 h were seeded and cultured for two weeks. The colonies were stained using crystal violet solution and then counted under a microscope. *** P < 0.001 (n = 3).

**Supplementary Fig. 4 USP9X is highly expressed and predicts poor clinical outcomes in lung cancer patients. a** Representative immunohistochemical staining for USP9X in lung cancer tissues and matched adjacent normal tissues. Scale bar, 10 μm. **b** Statistical analysis of IHC staining to detect the protein expression of USP9X in a lung cancer tissue microarray. **c** The Kaplan-Meier analysis indicates that high USP9X expression is correlated with adverse prognosis in lung cancer patients.

**Supplementary Fig. 5 Subcellular localization of endogenous KDM4C and USP9X in lung cancer cells.** Representative immunofluorescent images of KDM4C (red), USP9X (green) and DAPI (blue) in SPC-A1 cells. Scale bar, 10 µm.

| **Genes** | **Sequences (5'--3')** |
| --- | --- |
| TGF-β2 | F: TGAGTCACAACAGACCAACCG |
|  | R: CCATTTCCACCCTAGATCCC |
| BMP2 | F: GTGGGGTGGAATGACTGGAT |
|  | R: GAGATAGCACTGAGTTCTGTCGG |
| JUND | F: GACCCTCAAGAGTCAGAACACG |
|  | R: GTTGACGTGGCTGAGGACTT |
| GDF11 | F: CACCACCGAGACCGTCATTA |
|  | R: CTGGGCCTTCAGTACCTTTGT |
| BMP4 | F: CCAAGCGTAGCCCTAAGCAT |
|  | R: CTGAAGTCCACATAGAGCGAG |
| KDM4C | F: ACTCTGCTCATGCCGTACCA |
|  | R: CTTTCCCTCCGATGTAACGAC |
| GAPDH | F: GAGTCAACGGATTTGGTCGT |
|  | R: GACAAGCTTCCCGTTCTCAG |

F, forward primer; R, reverse primer.

**Supplementary Table 1** Sequences of primers used for Real-time quantitative PCR.

| Genes | Primer | **Sequences (5'--3')** |
| --- | --- | --- |
| TGF-β2 | Primer 1 | F: TTTTGCTACTCTATCCAC |
|  |  | R: CAAACCAGAGCTACTGAG |
|  | Primer 2 | F: AGCTTTGCTTCAACTCAA |
|  |  | R: TCCTCATTCTGGAACATT |
|  | Primer 3 | F: ATTCCTGTTTGTCAGAAC |
|  |  | R: ACTACACTTAGTAATTTG |
|  | Primer 4 | F: TAACCTAGGCCCCATACA |
|  |  | R: ACTGTTCCACCTTCCCTT |
|  | Primer 5 | F: CACTTAAAATTCCAGCTT |
|  |  | R: TTTACAATGTACCCCTCT |
|  | Primer 6 | F: AAGATTGGAAGGTATGTC |
|  |  | R: AAGGAGGTGGAATGTGCG |
|  | Primer 7 | F: CTCGTGGTCTAAGTAACG |
|  |  | R: CCTGCCTCTTTCACTTGC |
| BMP2 | Primer 1 | F: TTATGTAAGTTGTGCTTG |
|  |  | R: ATATCCTGTGATGACCTC |
|  | Primer 2 | F: TTGAGGTCATCACAGGAT |
|  |  | R: GGAGGCAATAAAACATAT |
|  | Primer 3 | F: CGTGTCACAGCCCTACTT |
|  |  | R: TGTGGCAGACTGAAGGAT |
|  | Primer 4 | F: CACGTAAAGCAGGATGAC |
|  |  | R: AGGCTGGAAATTGAGTGT |
|  | Primer 5 | F: CCCTGACCCTGCATTTGTCC |
|  |  | R: TAAAGGGGACGCCGCCTG |
| JUND | Primer 1 | F: CGTCCTTCTTCATCATGCTGCC |
|  |  | R: GGGAGGCGGAGGATGGAAAC |
|  | Primer 2 | F: GGCTGATGAGGCAGGAAT |
|  |  | R: CATGCAGAGGCTGTAGGT |
|  | Primer 3 | F: GTCTGAATGGAGCCCACA |
|  |  | R: CATAGGCAATGTCCCTGA |
|  | Primer 4 | F: GCTGAGATTGTGCCACTG |
|  |  | R: GAGGACAGCCTCCCTTAT |

F, forward primer; R, reverse primer.

**Supplementary Table 2** Sequences of primers used for Chip-PCR.
