# Supplementary figures and images for: USP9X-mediated KDM4C deubiquitination promotes lung cancer radioresistance by epigenetically inducing TGF-β2 transcription
Source: Cell Death Differ. 2021 Feb 8;28(7):2095–111. doi: 10.1038/s41418-021-00740-z (PMC8257660; doi:10.1038/s41418-021-00740-z)

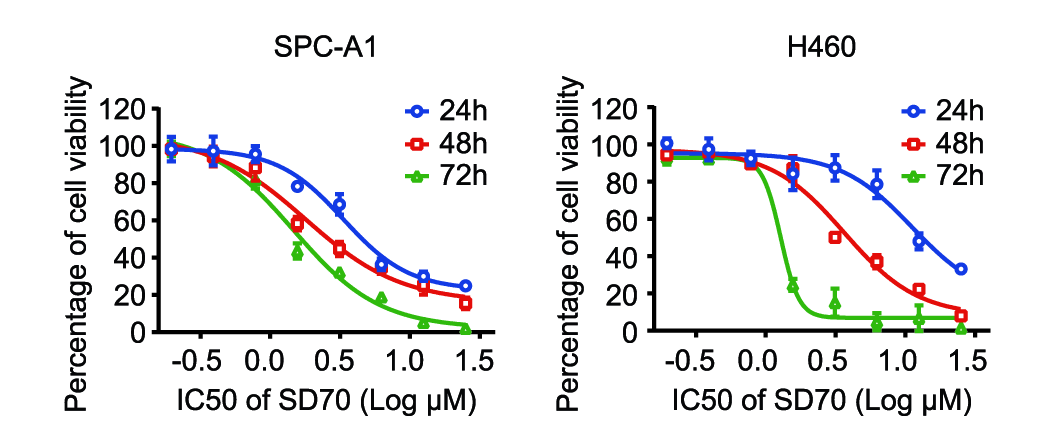

Supplement: Supplementary file 2 — Supplementary Figure 1 [file 41418_2021_740_MOESM2_ESM.tif]

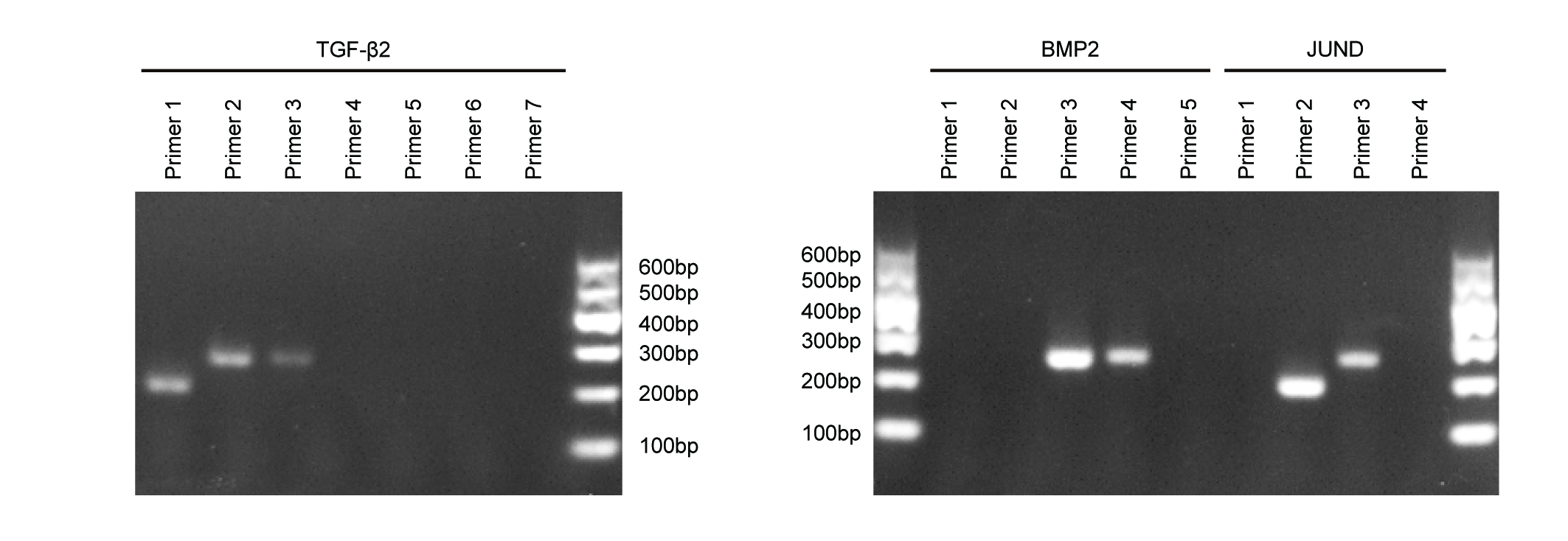

Supplement: Supplementary file 3 — Supplementary Figure 2 [file 41418_2021_740_MOESM3_ESM.tif]

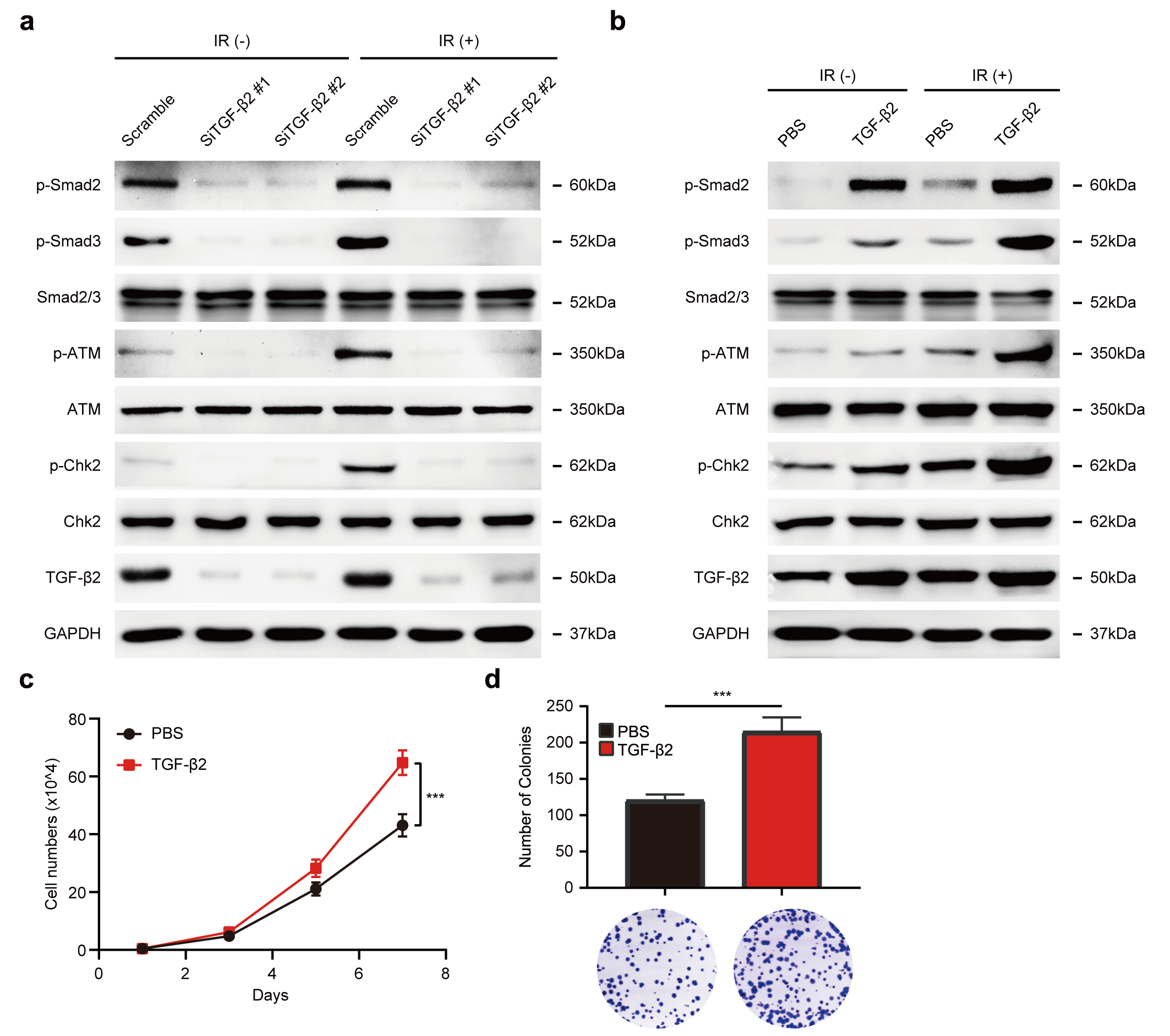

Supplement: Supplementary file 4 — Supplementary Figure 3 [file 41418_2021_740_MOESM4_ESM.tif]

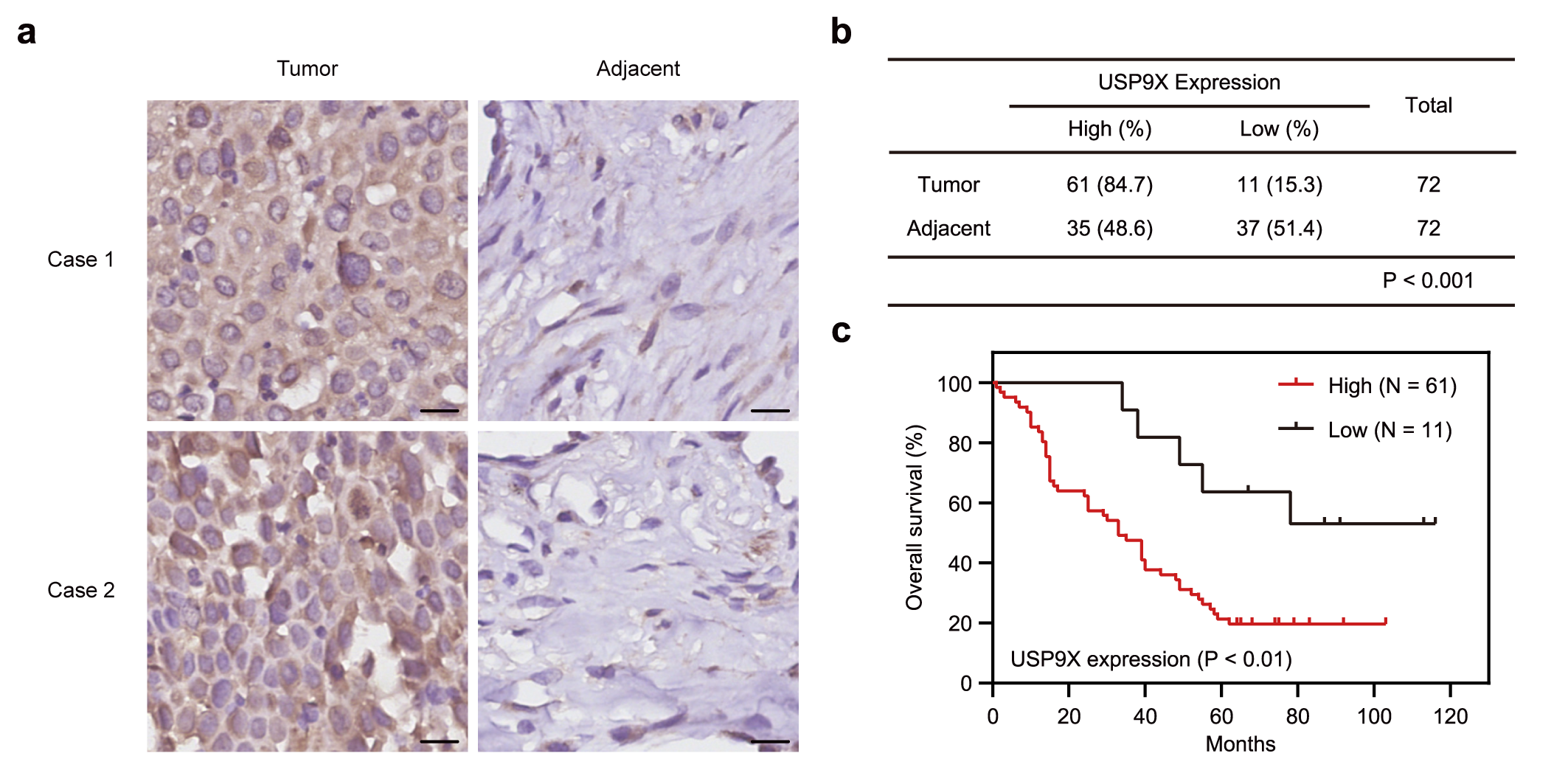

Supplement: Supplementary file 5 — Supplementary Figure 4 [file 41418_2021_740_MOESM5_ESM.tif]

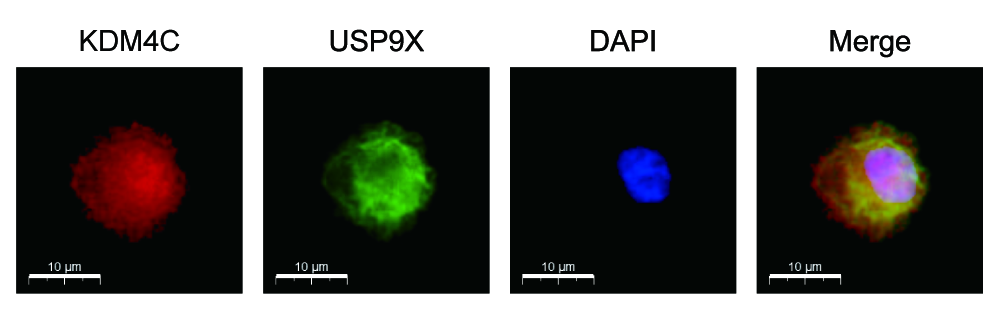

Supplement: Supplementary file 6 — Supplementary Figure 5 [file 41418_2021_740_MOESM6_ESM.tif]
